# Arc Jet Testing and Modeling Study for Ablation of SiFRP Composites in Shear Environment

**DOI:** 10.3390/ma18174142

**Published:** 2025-09-04

**Authors:** Meicong Wang, Jixiang Shan, Xin Yang, Qianghong Chen, Yonggang Lu, Yupeng Hu

**Affiliations:** Institute of Systems Engineering, China Academy of Engineering Physics, Mianyang 621900, China; wmc714@163.com (M.W.);

**Keywords:** silica-reinforced composite, ablation, shear environment, carbon deposition, surface recession, thermal response

## Abstract

The ablation process of a silica fiber-reinforced polymer (SiFRP) composite under aerodynamic heating and a shear environment was investigated by experiments and numerical study. The flat plate samples were tested in an arc jet wind tunnel under heat flux and pressure ranging from 107 W/cm^2^ at 2.3 kPa to 1100 W/cm^2^ at 84 kPa. The heating surface experiences shear as high as 1900 Pa. The in-depth thermal response and ablating surface temperature of the specimens are measured during ablation. According to the ablation experimental results, a multi-layer ablation model was established that accounts for the effects of carbon deposition, investigating the thermophysical properties of the ablation deposition layer. The accuracy of the proposed ablation model was evaluated by comparing the calculated and experimental surface ablation recession and internal temperature of a silica–phenolic composite under steady-state ablation. Carbon–silica reaction heat is the important endothermic mechanism for silica-reinforced composites. The research provides valuable reference for understanding the ablative thermal protection mechanism of silicon–phenolic composites in a high shear environment.

## 1. Introduction

As a typical ablative thermal protection material, SiFRP composites have advantages such as a relatively low thermal conductivity, resistance to oxidation, mature manufacturing process, and low cost. It is commonly used in thermal protection systems for planetary entry vehicles, engine nozzles, etc. [1,2,3,4,5].

As the material temperature gradually increases, the main ablation processes [6,7] of silica–phenolic resin include the following: pyrolysis of phenolic resin, melting of silica, flow and evaporation of melt, carbon–silicon reaction, carbon oxidation, etc. Ablation models that account for all these processes are highly complex. An ablation model based on a liquid layer [6,8,9,10] generally ignores the heat entering the material and is suitable for characterizing stagnation ablation under a severely high heat flux and negligible shear environment. In fact, thermal decomposition significantly increases the thermal conductivity of the charred material compared to virgin material [11,12,13]. The effect of phenolic resin pyrolysis on the thermal properties of SiFRP composite materials under a high heating rate was studied [14]. Therefore, the heat entering the interior of the material increases [15,16,17], leading to a more pronounced thermal response in the internal material and further inward penetration of thermal decomposition. A multi-layer material ablation model [11,18,19] including a liquid layer, char layer, pyrolysis layer, and virgin layer provides a comprehensive approach for studying the surface ablation and volume ablation of materials. The model accounts for the heat and mass transfer of charred materials during decomposition and surface recession with pyrolysis gases escaping from the high-porosity structure of the char layer. Also, the cooling effect on the char and the injection into the boundary layer of pyrolysis gases have a significant impact on the ablative behaviors in the model. In a shear environment, the measured recession of ablative materials is higher than the predicted results from general models, and one possible factor leading to this discrepancy is the flow of the melt layer (off the model) [20,21,22]. So, the effect of the melt flow on surface retreat should be included in the ablation model.

Carbon deposition has been observed beneath the front surface during the ablation of silica–phenolic composites [12,23]. The phenomenon is relative to the chemical vapor deposition from polymer pyrolysis gases, which contain significantly more carbon than in equilibrium states [24,25]. Carbon deposition reduces the pore size of the char, leading to a densification of the structure [23,26]. Therefore, deposited carbons increase the density and thermal conductivity of the char layer. A carbon–silica reaction that occurs at high temperatures in silica–phenolic composites is a highly endothermic reaction [27,28,29]. The reaction plays an important role in absorbing heat during the ablation process and can be crucial in determining the effectiveness of ablative resin systems. In fact, carbon deposition increases the mass fraction of carbon in the char, promoting the occurrence of this endothermic reaction. Therefore, it is necessary to characterize the thermophysical properties and the endothermic reaction of carbon deposition materials within the ablation model.

The ablation tests of SiFRP material under strong shear conditions were performed using arc jet experiments. An ablation model incorporating the carbon deposition layer was developed referencing multi-layer ablation models for charred materials. The thermophysical parameters of the carbon deposition region were determined. The improved ablation model can better predict the ablation retreat and internal thermal response of silicon–phenolic composites under conditions of a medium heat flux density and strong aerodynamic shear forces. This study provides a reference for the design optimization and performance prediction of silicon–phenolic thermal protection systems in similar environments.

## 2. Ablation Test Methods and Typical Experimental Results of SiFPR Composites

**Figure 1:**
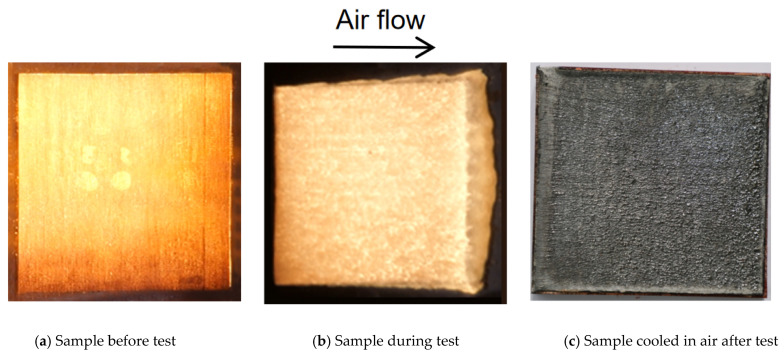
Flat plate sample of SiFRP composites before, during, and after test.

Figure 1a shows a flat plate model of the SiFRP composites used in the arc jet test. The sample size before the test is 100 mm in length, 100 mm in width, and 15 mm in thickness. The samples are first pre-formed by impregnating silica fabric (SiO_2_ content ≥ 96 wt.%) with phenolic resin, followed by polymerization and final mechanical processing. The silica and phenolic resin in the composites are about 67 wt.% and 33 wt.%, respectively (measured in a semi-dry state after impregnation and heating pretreatment). The properties of the solid silica fiber are as follows: density of 2150 g/cm^3^, melting temperature of about 1700 °C, and evaporation temperature of about 2230 °C at an atmospheric pressure.

Some test specimens were equipped with a thermocouple plug. Four thermocouples were arranged in the plug at different depths, designated as TC01, TC02, TC03, and TC04, with the nominal distances from the original heating surface of the sample 0 mm, 3 mm, 6 mm, and 11 mm, respectively. The measurement error was ±4 °C or ±1% of the measured value when the temperature was among −20 °C~400 °C or greater than 400 °C, respectively.

**Figure 2:**
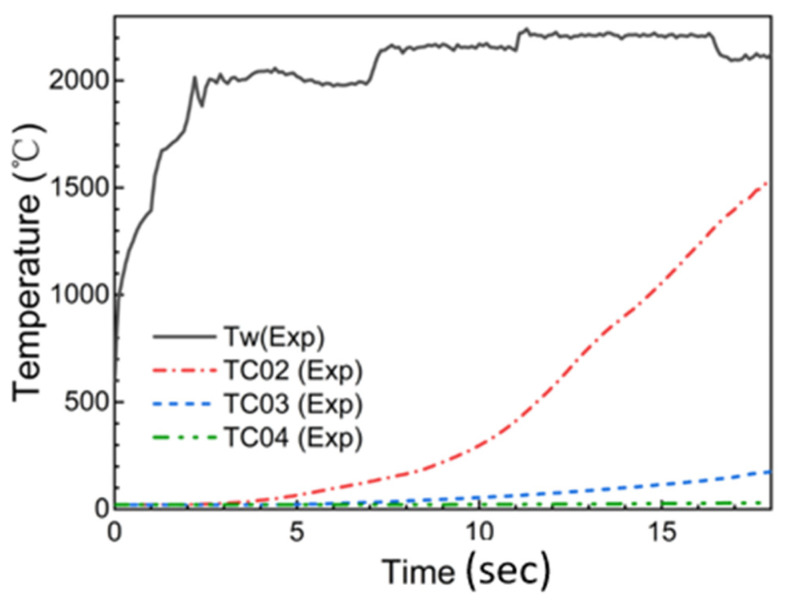
Surface and in-depth temperature of test sample during ablation.

Ablation tests were carried out using supersonic turbulent plate ablation test equipment (China aerodynamics research and development center, Mianyang city, China) [30]. The heating surfaces of the specimens were at a small angle to the direction of the flow. The heat flux and pressure of the sample surface ranged from 107 W/cm^2^ at 2.3 kPa to 1100 W/cm^2^ at 84 kPa during the arc jet test. The heating surface experiences shear as high as 1900 Pa. The surface material melted under aerodynamic heating. Then, the molten materials flowed and formed a flowing liquid layer covering the heated surface gradually under the action of external aerodynamic shear force, as shown in Figure 1b. Flow led to the mass loss of molten material and surface recession. A colorimetric pyrometer was used to measure the surface temperature of the sample. The surface temperature and thermocouple measurement data of the two samples with the thermocouple plug installed are shown in Figure 2. At 7 s from the beginning of the experiment, a balance was reached at a surface temperature of 2150 °C between the aerodynamic heating, material ablation heat, external radiation heat, and material heat absorption. Then, the surface temperature rose to 2200 °C at 11 s. There was a significant temperature rise at 3 mm in depth and a slight rise at 6 mm in depth during ablation. And the temperature at 10 mm in depth remained essentially unchanged all the time.

**Figure 3:**
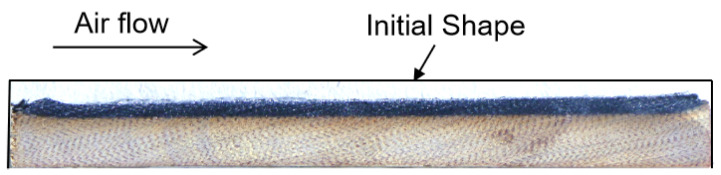
Cross-section of the flat plate sample after the test (along the air flow direction).

The appearance of the flat specimen cooled in air after ablation is shown in Figure 1c. To understand the volume ablation of the material, the ablated specimens were dissected along the air flow direction. Figure 3 shows a cross-section of the specimen after the test. The cross-section is 15 mm distance from the center of the heated surface. The black line indicates the initial shape of the specimen before the test. As shown in Figure 3, the ablated sample exhibits clear layering: the outermost surface is a thin layer of molten material, the black area beneath is the charred material, and the bottom region consists of the original material. The sample was imaged using a 10× optical microscope.

An optical microscope was used to image the cross-section of the tested sample, and then the surface ablation retreat can be measured. The surface ablation recession was measured to be between 2.76 mm and 2.93 mm, with an average value of 2.85 mm. The average depth of pyrolysis proceeding inside the sample was approximately 4.96 mm.

**Figure 4:**
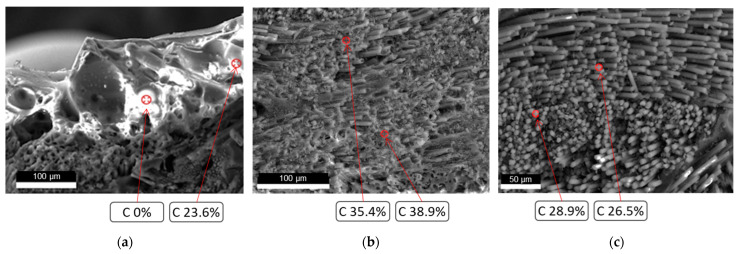
In-depth microstructures and carbon contents of tested silica–phenolic composites. (**a**) Silica melt with a large number of voids (ablated surface). (**b**) Carbon deposition region with dense structure (1 mm depth from ablated surface). (**c**) Pyrolysis layer with a fiber structure and high porosity (2 mm depth from ablated surface).

An electron microscope was used to image the cross-section and microstructure of the sample. The representative microstructures at different depths of the cross-section obtained by electron microscopy are shown in Figure 4. The liquid layer is composed of molten material and a large number of pores, as shown in Figure 4a. A dense structure with a thickness of about 1 mm appeared below the molten. EDS analysis shows that the carbon mass fraction in the dense layer is much higher than that in other regions, corresponding to the carbon deposition regions. Below the dense layer is a fiber structure with high porosity, which corresponds to the pyrolysis layer.

**Figure 5:**
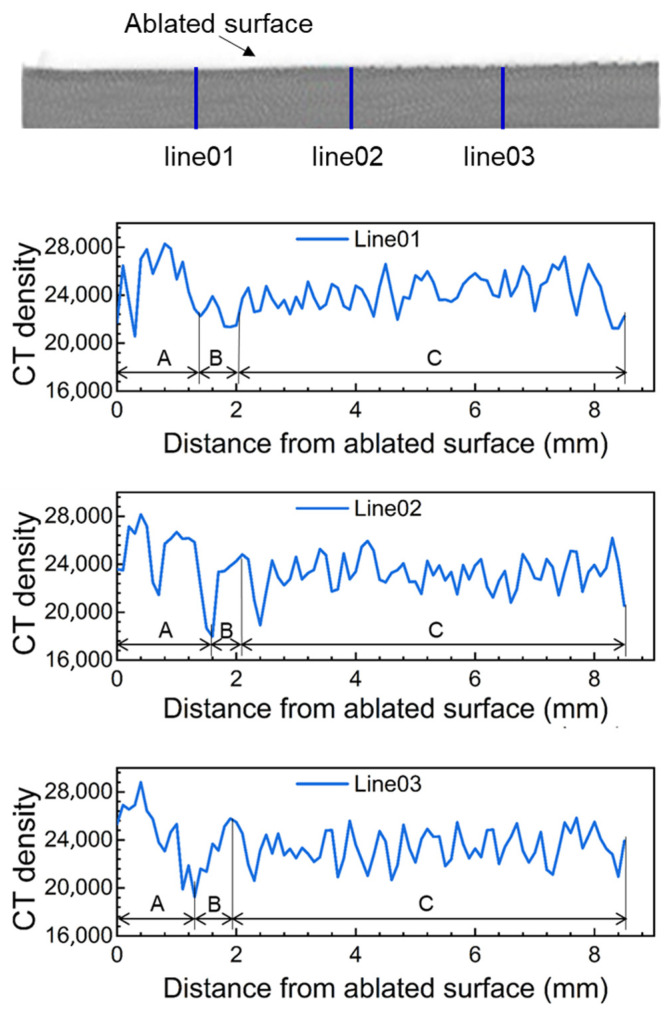
CT density of the tested sample along ablation retreat direction.

To understand the density characteristics of carbon deposition regions, the CT density of the sample after the test was obtained by computed tomography (CT). The CT values are dimensionless grayscale values of the specimen. For silica–phenolic composites, the CT density is approximately proportional to the physical density [31]. Figure 5 shows the CT density along three lines located at different positions in the direction of the ablation retreat of the specimen after ablation. The variation in the CT density on the same line can characterize the density of materials at different depths from the ablation surface. Due to the difference in density between resin and silica fibers, the CT density values of composite materials fluctuate at different positions. According to the variation characteristics of the CT density values, the line can be divided into three regions: A, B, and C.

The average CT density of each region is shown in Table 1. The peak density of area A is significantly higher than other areas, corresponding to the carbon deposition layer in Figure 4. In each line, the CT density value of area A increases with the decreasing depth. When approaching the ablated surface, the CT density decreases because some carbon is consumed due to oxidation reactions. Along the direction of the air flow, the average CT density of area A in the upstream area is larger than that in the downstream area. One possible factor is the higher surface pressure; thus, there is more pronounced compression of the charred material. The CT density of area B decreases with depth, corresponding to the pyrolysis layer of Figure 4. Due to the escape of pyrolytic gases, the minimum CT density in the area is significantly lower than that in other regions. The CT density values of area C, corresponding to the virgin material, remain relatively stable compared to regions A and B.

## 3. Ablation Model

**Figure 6:**
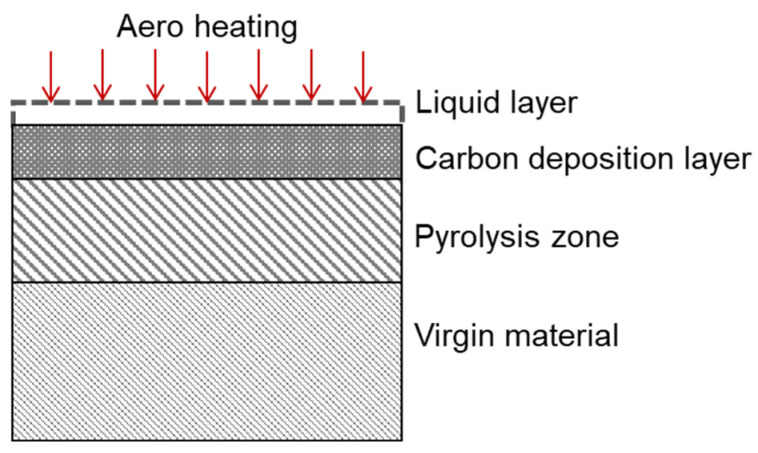
Multi-layer ablation model of SiFRP materials.

Based on the classic liquid layer model and multi-layer ablation model, a carbon deposition layer was introduced into the ablation model, as shown in Figure 6. The model includes a liquid layer, carbon deposition layer, pyrolysis layer, and original layer. The carbon deposition effect is included in the ablative performance of SiFRP materials. Based on the ablation test results of the SiFRP material above, the thermal properties of this layer of material were determined.

### 3.1. Surface Ablation Model

Surface ablation processes mainly include pyrolysis, a carbon–silica reaction, carbon oxidation, material melting, and melt evaporation. When the surface of a material begins to retreat, the physical or chemical processes that cause material mass loss include liquid layer flow, a carbon–silica reaction, carbon oxidation, evaporation, etc. The mass loss caused by evaporation is negligible because the wall temperature is lower than the sublimation of silica fiber. The mass conservation equation of the ablated surface is shown in Equation (1).

(1) $${\dot{m}}_{-\infty }={\dot{m}}_{\tau ,L}+{\dot{m}}_{p,L}+{\dot{m}}_{R,L}+{\dot{m}}_{CO,L}$$

where ${\dot{m}}_{-\infty }$ is the total mass loss rate during surface ablation, and the subscript L represents the liquid layer. The four terms on the right-hand side give the mass loss rate of the liquid layer due to external force loading (${\dot{m}}_{\tau ,L}$), pyrolysis (${\dot{m}}_{p,L}$), carbon–silica reaction (${\dot{m}}_{R,L}$), and carbon oxidation (${\dot{m}}_{CO,L}$), respectively.

Equilibrium ablation is a good assumption for surface temperatures of 2000 K or greater for a carbonaceous material [17]. When using the wall temperature as the boundary, the energy conservation model for the ablative wall does not need to include evaporative heat and radiative heat. Equation (2) shows the simplified energy conservation equation on the gas–liquid interface.

(2) $$Ak_{L}\nabla T(x,t){|}_{x=x_{W}}={\dot{m}}_{\tau ,L}C_{p,L}\left(T_{w}-T_{0}\right)+{\dot{m}}_{p,L}\Delta h_{p}+{\dot{m}}_{R,L}\Delta h_{R}-{\dot{m}}_{CO,L}\Delta h_{CO}+q_{N}$$

where *A* is the ablated surface area; *T*, *C*p, *k*, and h are the temperature, specific heat, thermal conductivity, and heat of the reaction, respectively. ∇T(x,t) is the temperature gradient in the ablation direction. The subscripts w and 0 represent the ablated wall and initial material, respectively. The term on the left-hand side gives the heat conducted through the gas–liquid interface. The five terms on the right-hand side are, respectively, related to the heat sink of the ablated mass loss due to external force loading, thermal decomposition, carbon–silica reaction, oxidation reaction of the char, which is exothermic, and net heat flux transferring into the internal material.

### 3.2. Volumetric Ablation Model

According to the material temperature, the internal ablation reactions mainly include pyrolysis and a carbon–silica reaction. The solid phase has less contact with oxygen in the gas boundary layer as the surface covered with viscous molten, and the influence of carbon oxidation can be ignored. The effects of carbon deposition on the ablation model mainly include the increased char density and thermal conductivity, and the endothermic reaction between the deposited carbon and silica. Considering that the thickness of the carbon deposition layer in the sample is relatively small, the effects of the increasing density and thermal conductivity are relatively minor, while the influence of the endothermic reaction between the deposited carbon and silica dominates.

#### 3.2.1. Mass Conservation Equation Including Carbon Deposition

The mass variation rate of a control volume in the solid phase can be expressed in terms of the mass loss rate due to pyrolysis (${\dot{m}}_{p,s}$), a carbon–silica reaction (${\dot{m}}_{R,s}$), and carbon deposition (${\dot{m}}_{d,s}$) as follows:

(3) $$\left\{\begin{array}{l} {\dot{m}}_{s}={\dot{m}}_{d,s}-{\dot{m}}_{p,s}-{\dot{m}}_{R,s},\;\;if\;\;T_{m}>T\;>\;T_{d,1}\\ {\dot{m}}_{s}=-{\dot{m}}_{p,s}-{\dot{m}}_{R,s},\;if\;\;T_{d,1}>T\;>\;T_{p} \end{array}\right.$$

where the subscript *s* represents the solid phase.

It is assumed pyrolysis gas flows and deposits along the thickness direction. The density of carbon deposition increases approximately linearly with the temperature [32]. The average density of the deposition area (${\bar{\rho }}_{d}$) among the deposition temperature range (*T*_d,1_, *T*_d,2_) is given as follows:

(4) $${\bar{\rho }}_{d}=\rho_{p}+k_{d}(T-T_{d,1}),\mathrm{if}\;T_{m}\;>\;T\;>\;T_{d,1}$$

where *ρ_p_* is the density of the pyrolysis products, and *k*_d_ is the linear increase slope of the density with the temperature.

Resin pyrolysis follows the Arrhenius law [12] shown in Equation (5).

(5) $$\frac{d\alpha }{dT}=\frac{A_{1}}{\beta }{\left(1-\alpha \right)}^{n_{1}}e^{-\frac{E_{1}}{RT}}$$

where *α* is the pyrolysis conversion rate equal to (*m*_0_ − *m*)/(*m*_0_ − *m_f_*), *β* is the heating rate, and *A*_1_, *n*_1,_ and *E*_1_ are the pre-exponential factor, order of reaction, and activation energy of pyrolysis, respectively. The pyrolysis kinetic parameters, shown in Table 2, are obtained based on the thermogravimetric analysis (TGA) data of phenolic resin at heating rates (5 K/s, 20 K/s, and 50 K/s) [12], using Friedman et al.’s conversion rate method [33].

The kinetics of high-temperature carbon–silica reactions for a silica–phenolic resin is shown as Equation (6) [29].

(6) $$-\frac{1}{m_{0}}\times \frac{dm}{dt}=A_{2}{\left(\frac{m-m_{f}}{m_{0}}\right)}^{n_{2}}e^{-\frac{E_{2}}{RT}}$$

where *m*_0_, *m_f_*, and *m* are the original mass before the reaction, the final mass after the reaction, and the instantaneous mass, respectively; *A*_2_ and *E*_2_ are the pre-exponential factor and activation energy of the carbon–silica reaction, respectively.

#### 3.2.2. Energy Conservation Equation Including Carbon Deposition

The energy conservation equation of a control volume inside the matrix is taken as Equation (7), when the convective cooling effect of the pyrolysis gas is ignored.

(7) $$\left\{\begin{array}{l} \frac{\partial }{\partial x}\left[k\frac{\partial T}{\partial x}\right]={\dot{m}}_{p,s}\Delta h_{p,s}+{\dot{m}}_{R,s}\Delta h_{R,s}+A\rho C_{P}\frac{\partial T}{\partial t}\Delta x+q_{N,out},\;\;if\;\;T_{m}>T>T_{R}\\ \frac{\partial }{\partial x}\left[k\frac{\partial T}{\partial x}\right]={\dot{m}}_{R,s}\Delta h_{R,s}+A\rho C_{P}\frac{\partial T}{\partial t}\Delta x+q_{N,out},\;if\;\;T_{R}>T>T_{p} \end{array}\right.$$

here *T*_p_, *T*_R,_ and *T*_m_ are the pyrolysis temperature, carbon–silica reaction temperature, and melting temperature of silica fiber; *k*, *C*_p_, and *ρ* are the thermal conductivity, heat capacity, and density, respectively; and *h*_p,s_ and *h*_R,s_ are the heat of the decomposition and carbon–silica reaction. The terms on the left-hand side of the equations give the heat conducted through the boundary of the control volume. The four terms on the right-hand side of Equation (7) are, respectively, related to the thermal decomposition, carbon–silica reaction, heat sink, and net heat flux transferring into the control volume.

The effective thermal conductivity (${\bar{k}}_{d}$) is shown as Equation (8), while the pyrolysis products and deposited carbon are assumed to be in parallel in the deposition area for heat transfer.

(8) $${\bar{k}}_{d}=k_{p}+{k_{car}}^{f_{v,car}}$$

where *k*_p_ and *k*_car_ are the thermal conductivity of the pyrolysis products and deposited carbon, respectively, and *f*_v,car_ is the volume fraction of the deposited carbon.

## 4. Analysis

The surface ablation recession rate and in-depth thermal response can be comprehensively numerically calculated by combining the mass conservation Equations (1) and (3) and the equation of energy conservations (2), (7), using the outer wall temperature of the liquid layer as the boundary. The calculation is simplified based on the following assumptions:

1. The thickness of the liquid layer is assumed to be constant.
2. The specific heat, density, and thermal conductivity of the melt are assumed to be constant in the analysis, although they vary somewhat with the temperature. The effective thermal conductivity and density of the liquid layer are assumed to be linearly correlated with the porosity.
3. The aerodynamic shear force acting on the liquid layer is assumed to be great enough, so that the liquid phase transformed from the solid phase instantly leaves its original position. The melting temperature of the silica fibers is 1700 °C.
4. The thermal expansion due to pyrolysis is neglected since the thickness of the charred layer is small.

**Figure 7:**
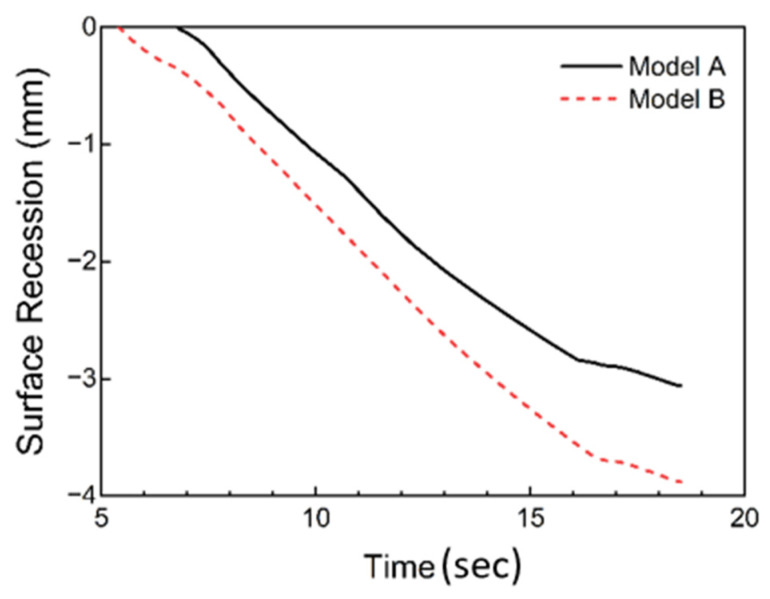
Comparison of calculated and experimental surface recession for silica–phenolic composites.

The surface recession process calculated by our presented ablation model (named “Model A”) is shown in Figure 7. The total surface recession predicted by Model A is 3.06 mm, which is about 7% higher than the experimental result. As a comparison, the simulation results from the ablation model without carbon deposition (named “Model B”) are also provided. The retreat amount of Model B is 30% higher than the experimental results. Moreover, the surface temperature of the solid matrix in Model B needs to be at an unreasonable value far above the melting temperature during steady ablation. The prediction results of Model A are more consistent with the experimental results compared with Model B.

**Figure 8:**
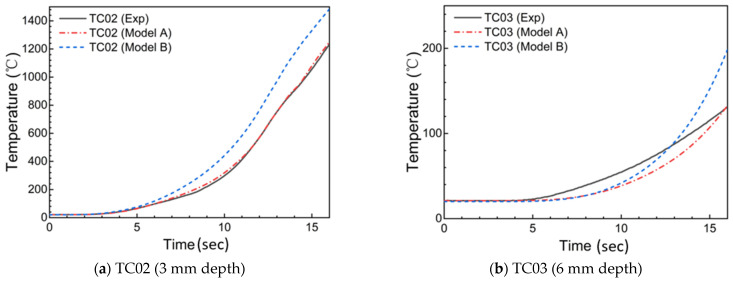
Predicted and experimental in-depth temperatures for silica–phenolic composites under shear environment.

The experimental and predicted results for the thermal response of the internal material are shown in Figure 8. The in-depth temperatures calculated by Model A are more consistent with the experimental results than those calculated by Model B. For the 3 mm and 6 mm in-depth temperatures, errors between the simulation results of Model A and the experimental results are approximately 12% and 40%, respectively. The errors are due to factors such as non-uniformity of the composite materials, measurement errors of the thermocouples, etc.

**Figure 9:**
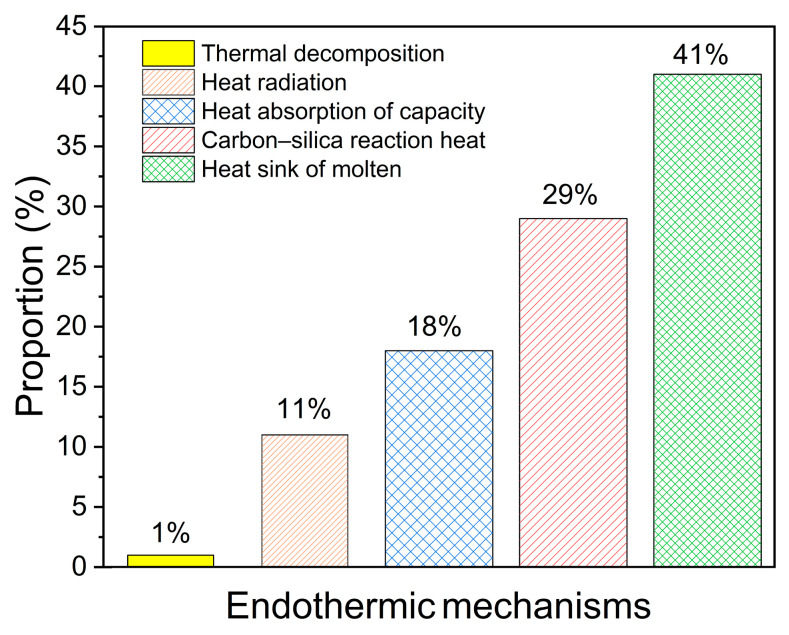
The proportion of the heat absorption induced by each endothermic mechanism in the total heat absorption of SiFRP composites under shear environment.

Figure 9 shows the proportion of the heat absorption induced by each endothermic mechanism in the total heat absorption of silica–phenolic composites under a shear environment. As can be seen, the heat sink of molten lost under external force is the main endothermic mechanism for silica-reinforced composites, which makes up 41% of the total heat absorption. The carbon–silica reaction heat, heat absorption of capacity, heat radiation, and thermal decomposition take 29%, 18%, 11%, and 1% of the total heat absorption, respectively.

Carbon deposition slightly increases the thermal conductivity and heat capacity of the carbon layer while significantly increasing the proportion of the carbon–silica reaction heat in the total ablation heat. In general, carbon deposition enhances the latent heat of the ablation of silicon–phenolic materials. Therefore, it is effective to enhance the carbon deposition effect to improve the thermal protection performance of SIFRP composites under strong aerodynamic force.

## 5. Conclusions

Ablation tests of SiFRP composites were conducted in an arc jet wind tunnel under medium heat flux density and strong shear conditions. Based on the experimental results, a carbon deposition layer was introduced into the multi-layer ablation model. The thermal physical property model of the carbon deposition layer was studied. The main conclusions derived from the ablation tests and models are as follows. (1) The main reasons for surface ablation recession are the melting of surface materials due to aerodynamic heating, and the flow of molten off the model due to the shear force. (2) The molten materials are assumed to immediately leave their original position in the solid phase due to aerodynamic shear force; the surface ablation recession predicted by the proposed ablation model is 7% higher than the experimental results under steady-state ablation. (3) Carbon–silica reaction heat is the important endothermic mechanism for silica-reinforced composites, which contributes 29% of the total heat absorption.

## Figures and Tables

**Table 1:** Average CT density of each area.

	Average CT Density of Area A	Average CT Density of Area B	Average CT Density of Area C	Average CT Density of All Areas
Line01	26,299	22,505	24,227	24,254
Line02	24,211	22,868	23,342	23,511
Line03	23,884	23,326	23,409	23,594

**Table 2:** Properties of SiFRP composite materials.

Property	Value	Source
Density of virgin material, ρ_vir_ (kg/m^3^)	1640	Manufacturer (China space sanjiang group CO., LTD., Xiaogan, China)
Thermal conductivity of virgin material (room temperature to 250 °C), k_vir_ (W/m K)	0.4–0.7	
Specific heat capacity of virgin material (room temperature to 250 °C), C_pvir_ (J/kg K)	≥800	
Coefficient of thermal expansion of virgin material (room temperature to 250 °C), β_vir_ (1/K)	12 × 10^−6^	
Density of char material, ρ_char_ (kg/m^3^)	1230	[7]
Thermal conductivity of char material (room temperature to 850 °C), k_char_ (W/m K)	0.955 + 8.42 × 10^−4^ *T* − 4.07 × 10^−6^ *T*^2^ + 5.35 × 10^−9^ *T*^3^	[7]
Specific heat capacity of char material (room temperature to 850 °C), C_pchar_ (J/kg K)	1120.8 + 1.025 *T*	[7]
Density of molten layer, ρ_L_ (kg/m^3^)	2250	[6]
Thermal conductivity of molten layer, k_L_ (W/m K)	2.93	[6]
Specific heat capacity of molten layer, C_pL_ (J/kg K)	1046	[6]
Heat of pyrolysis, h_p_ (kJ/kg)	418	[1]
Average activation energy (phenolic resin pyrolysis), E_p_ (J/mol)	64,081	TGA
Pre-exponential factor (phenolic resin pyrolysis), A_p_ (1/s)	333	TGA
Order of reaction (phenolic resin pyrolysis), n_p_ (-)	1	TGA
Heat of carbon–silica reaction, h_R_ (kJ/kg)	5941	[6]
Average activation energy (carbon–silica reactions), E_R_ (J/mol)	353,758	[29]
Pre-exponential factor (carbon–silica reactions), A_R_ (1/s)	2.6167 × 10^−7^	[29]
Order of reaction (carbon–silica reactions), n_R_ (-)	0.532	[29]
Initial temperature of deposition, T_d,1_	1000 °C	[23]
End temperature of deposition, T_d,2_	1700 °C	Manufacturer (China space sanjiang group CO., Ltd., Xiaogan, China)

## Data Availability

The original contributions presented in this study are included in the article. Further inquiries can be directed to the corresponding author.
